# Inuit women's stories of strength: informing Inuit community-based HIV and STI prevention and sexual health promotion programming

**DOI:** 10.3402/ijch.v75.32135

**Published:** 2016-12-09

**Authors:** Jenny R. Rand

**Affiliations:** Interdisciplinary PhD Program, Faculty of Graduate Studies, Dalhousie University, Halifax, NS, Canada

**Keywords:** Inuit, community-based participatory research, HIV, prevention, sexually transmitted infections, sexual health, indigenous methodologies, Inuit Qaujimajatuqangit, Two-Eyed Seeing

## Abstract

**Background:**

There is a dearth of literature to guide the development of community-based HIV and sexually transmitted infection (STI) prevention and sexual health promotion programs within Inuit communities.

**Objective:**

The aim of this study was to create a dialogue with Inuit women to address the lack of information available to inform programming to improve the sexual health of Inuit women, their families, and their communities in the Canadian Arctic.

**Design:**

This study used Indigenous methodologies and methods by drawing from Inuit Qaujimajatuqangit and postcolonial research theory in a framework of Two-Eyed Seeing, and using storytelling sessions to gather data. Community-based participatory research principles informed the design of the study, ensuring participants were involved in all stages of the project. Nine storytelling sessions took place with 21 Inuit women aged 18–61 years. Storytelling sessions were audio recorded and transcribed verbatim, and Atlas.ti aided in the organization of the data for collaborative thematic analysis within three participatory analysis sessions with 13 of the participating women.

**Results:**

From the storytelling and analysis sessions, five major themes emerged: (a) the way it used to be, (b) change, (c) family, (d) intimate relationships and (e) holistic strategies. Participating women emphasized that HIV and STI prevention and sexual health promotion programming needs to take a holistic, community-wide, family-focused and youth-centred approach within their communities.

**Conclusion:**

Participants identified several important determinants of sexual health and shared ideas for innovative approaches they believe will work as prevention efforts within their communities. This article specifically focuses on key characteristics of programming aimed at STI and HIV prevention and sexual health promotion that were identified throughout participants’ stories. This study has provided a narrative to complement the epidemiological data that highlight the urgent need for prevention programming.

Inuit communities in the Canadian Arctic face unique challenges regarding HIV and sexually transmitted infection (STI) testing, treatment, prevention and care that include geographic location and cultural and linguistic obstacles for accessing these services (1). With these distinct characteristics of Inuit communities, there is a need for ongoing Inuit-specific sexual health promotion, education, disease prevention and care programs. Nunavut's rates of chlamydia, gonorrhoea and syphilis were reportedly 10 times higher than the Canadian national rate in 2013 (2). The Canadian Aboriginal AIDS Network contends that, with the exceedingly high rates of STIs, limited health care access and remoteness of communities, if no changes are made, rates of HIV in the North have the potential to increase dramatically (3,4).

Although data suggest HIV rates in Nunavut are currently low (2,5), there are many risk factors or indicators in the Northern populations that suggest HIV has the potential to spread quickly within Inuit communities. For example, Inuit are known to have a high birth rate, and with such high STI rates, both of these factors are associated with the risk of HIV infection via unprotected sexual intercourse. Public health officials are increasingly concerned about additional factors that increase Inuit community members’ exposure to HIV and hepatitis C infection, such as travel between northern communities and the Canadian south (6).

Colonization and westernization have been identified in the literature as contributing to the high rates of STIs within the Arctic (7,8). Changes resulting from colonization have weakened the traditional lines of communication, leaving parents and Elders (who were the primary educators of sexual and reproductive health in the past) feeling ill-equipped to teach their children about sexual health (9,10). In addition, evidence suggests that present-day familial relationships are influenced by the trauma of settlement and residential schools, resulting in an outstanding need for rebuilding relationships between Elders and youth to support parent–child communication on sexual health (11).

Given the immense societal changes within Inuit communities, and the influence these changes have had on sexual health, there is a need to better understand sexual health within Inuit communities to create more effective STI and HIV prevention and sexual health promotion programming. This article describes a study that explored the determinants of Inuit women's sexual health to inform the development of future programming for Inuit women, their families and their communities. The goal of this study was to establish a dialogue among Inuit women in the westernmost community in Nunavut about community-based HIV and STI prevention, as well as sexual health promotion programming. In doing so, this study sought to answer the following questions: What are women's perceptions of STIs/HIV in their community? What do women think would benefit the community in regard to sexual health? And what do women think are the determinants that most influence the sexual health of women and communities? This article specifically highlights findings that are useful in the development of future Inuit community-based STI and HIV prevention and sexual health promotion programming.

## Methodology

### Researcher location

The primary researcher of this study and author of this article is of settler ancestry. This research project took place within the community where her family has lived for more than 15 years. Because the primary researcher is a former resident of this community, this study is based on a well-established, long-term relationship that allowed for a collaborative research partnership that made this strengths-based research project possible.

### Research design

This study followed a community-based participatory research (CBPR) design (12,13), where community engagement commenced well before any recruitment or data collection. In the earliest stages of the research project, a community advisory committee was formed. Decisions were made collaboratively with the advisory community and primary researcher regarding data collection, honorariums, timelines, community involvement, means of communication, recruitment and applications for funding.

Before recruitment and data collection, this study received support from the Kugluktuk Hamlet Council and approval from the University of Victoria's Ethics Review Board, and a Nunavut Research License was obtained.

### Theoretical framework

The theoretical framework for this study was grounded in Two-Eyed Seeing (14,15) and drew on Inuit Qaujimajatuqangit (IQ) (16,17) and postcolonial research theory (18). Two-Eyed Seeing is a concept made known by Mi'kmaw Elder Albert Marshall and is a guiding principle for bringing together multiple worldviews (19) (for more on Two-Eyed Seeing, see 14,15,19). It was important that this research was grounded in local realities, and it thus required a theoretical framework relevant to participants. Two-Eyed Seeing provided the bridge between the Inuit ways of knowing (IQ) and the academic ways of knowing (postcolonial research theory), and it drew on the strengths of each.

IQ refers to Inuit worldview, “Inuit epistemology or the Indigenous knowledge of the Inuit” (20, p. 1). IQ informed many aspects of the initial design of the research project and was considered throughout all phases. For example, the participatory design of the project reflected the IQ principle of collaboratively working together for a common good known as Piliriqatigiingniq (21). The concept of Aajiiqatigiingniq, decision-making together by taking counsel and comparing points of view (21), was also drawn from in this project. For example, in the development of this research project, an advisory group was formed and consulted, as was the Hamlet's Senior Administrative Officer, the Mayor, Hamlet Council and the wellness committee. The advisory committee as well as research participants were involved the decision-making process related to data collection, analysis, timelines and knowledge translation.

Pijitsirniq, the concept of serving a purpose and serving your community as well as providing for family and community (21), was present in this research project on multiple levels. For example, pijitsirniq aligns with the long-term goal of this project of informing programming and policy with the aim of improving the health of the community. In addition, this principle may be related to the women's interest (or motivation) in sharing their knowledge and experiences as a contribution to improving the health of their families and community.

The concept of Ikpigusttiarniq, meaning “caring for others; taking situations and who they are into account” (21, p. 2), can be seen within the aim of the study to examine what determines sexual health and what diverse factors influence sexual health. Taking time to explore the aspects of people's diverse backgrounds, such as structural and environmental factors that are beyond them as individuals, considers each person's situation. The structure of this project created a space for participating women to share, heal and care for one another, and this reflected Ikpigusttiarniq.

This is not an exhaustive description, but rather provides a few examples of how IQ was drawn from and reflected throughout the study. Elsewhere in the literature, Healey and Tagak (22) present the *Piliriqatigiinniq Model for Public Health Research*, a model for community health research that is rooted in Inuit ways of knowing, including several of the IQ principles outlined above.

### Recruitment

Recruitment was conducted through the advisory committee, a community feast and information session, and by advertising for volunteers and sharing information at grocery stores, workplaces, sewing groups, Facebook and community programs. Purposive sampling was used with the aim of recruiting females over 18 years of age who lived in Kugluktuk and who were interested in talking about sexual health promotion and HIV and STI prevention. Another aim was to ensure that there were women from various age groups to guarantee diverse generational perspectives. In total, 21 women ranging in age from 18 to 61 years were recruited for storytelling sessions. All of the participating women were living in Kugluktuk and had either lived there all of their lives or for several decades. Their educational backgrounds varied from primary school education only to university graduates. Their employment status ranged from unemployed and on social assistance to employment with the Hamlet of Kugluktuk or with the Government of Nunavut.

### Data collection

Nine storytelling sessions took place, with 21 women in total. The storytelling sessions began by sharing food and tea. The sessions were semi-structured as guiding questions were read aloud to the participants. The guiding questions requested the participants share stories related to what they thought about STIs and HIV in their community, what they thought influences the sexual health of women and communities, and what they thought would benefit their community in regard to sexual health promotion and STI and HIV prevention. The sessions lasted between 30 and 75 min. The session group sizes ranged from one on one to groups of six. Storytelling sessions were audio recorded and took place in multiple settings including participant's homes, Elders’ Centre, Prenatal Nutrition Program meeting space and a boardroom.

### Data analysis

Audio recordings of the storytelling sessions were transcribed verbatim. Once identifying information was removed, the transcripts were imported into the qualitative data analysis software Atlas.ti. Data analysis began with the primary researcher reviewing the transcriptions within Atlas.ti and roughly coding the data, to aid in organizing the data into a manageable size. The roughly coded data were brought back to storytelling session participants to conduct participatory analysis. Three participatory analysis groups were held with 13 of the women who participated in the storytelling sessions. The codes were discussed, organized and then some were collapsed and categorized into themes. Major themes emerged as headings as the analysis groups examined the codes, declared themes and indicated how the themes were related to one another. This was done by using slips of paper labelled with each of the themes and organizing them as they were seen to be related. Inductive thematic analysis was used as this project aimed to gain knowledge to inform future policy and prevention programs, which matches the output of applied thematic analysis (23).

## Findings and discussion

Five major themes and 27 subthemes emerged from the storytelling sessions and participatory data analysis. These major themes included (a) the way it used to be, (b) change, (c) family, (d) intimate relationships and (e) holistic strategies. Table I shows the major themes, subthemes and nested themes in their entirety. These findings revealed the participating women's perspectives on STI, HIV, community sexual health, key determinants of sexual health and innovative prevention approaches for their community. A selection of themes and quotes are briefly presented below and focus on key components of HIV and STI prevention and sexual health promotion identified by the participating women.

**Table I:** Major themes and subthemes with nested themes

Major theme	Subthemes and nested themes (•)
The way it used to be	Elders’ teachings
	Rules and order
	Arranged marriages
	Multiple spouses and swapping
	Menstruation
Change	Sexual health teachings
	Alcohol use
	Sex exchange
	Gender and power
	Transience and travel
	Taboo
Family	I tell my children
	Future generations
	Family homes
Intimate relationships	Communication
	Self-esteem and self-image
	Role modelling
	Incest
Holistic strategies	Awareness and information
	Message delivery
	Responsibility
	• Parents
	• Schools
	• Hamlet
	• Health system
	Continuity of community health care
	Testing
	More support
	• Contracting STIs
	Reaching the hard to reach
	• Dropouts/non-attenders
	• Men
	Substance use and sexual decision-making
	Condoms

The first major theme, the way it used to be, is made up of five subthemes (see Table I) and is named for the phrase that started many of the stories shared by women. The participating women began their stories referring to *the way it used to be* to honour and recognize the teachings and the ways of the past without judgement. The following quote from the subtheme *Elders’ teachings* indicates that historically, STIs such as HIV did not exist among Inuit, thus Elders have little knowledge on the subject, suggesting that information about HIV would be missing from their teachings:

But for things like that Inuit people or Elders don't talk about HIV because they didn't have that sickness until not too long ago. It might be long ago but some Elders really didn't know anything what was HIV and the sickness. The only time they found out what was HIV and when the health centre spread the word. (Storyteller, age 50)

Elders’ teachings create strong linkages throughout communities and families. Inuit children traditionally learned by carefully observing and following their Elders (24). Elders do not necessarily have the knowledge about STIs and HIV, which suggests a gap in knowledge translation that has taken place. Given that traditionally Inuit learned by observing and following their Elders, with HIV and STI information, this link is missing. This gap was identified by Pauktuutit Inuit Women of Canada, who have recently developed an Inuit sexual health glossary of terms to aid in communication and comprehension about sexual health, as many of the sexual health concepts and terms did not exist within Inuit dialects (see Tukisiviit – Do you understand? (25)). Elders have an important role in community sexual health and are what ties these three elements of community-wide, family-focused and youth-centred approaches together. Indigenous community-based HIV research has shown that Elder involvement is imperative for fostering relationships and ensuring respect and culturally safe and Indigenous ways of knowing are upheld in the research process (26).

The second major theme, change, was made up of six subthemes (see Table I). *Alcohol use* was the most salient subtheme emerging from all nine storytelling sessions. Stories of alcohol use were often the first thing women discussed in response to what they thought contributed to negative sexual health outcomes. This subtheme sits within the major theme of change, as the introduction of alcohol to Northern communities resulted in a major change in the way people behaved and related to one another. Reflecting on her memory of when alcohol was introduced to her community one woman shares,

I remember when I was a kid and all the alcohol and that came to the DEW* lines, that's when … it's like I got so lost. I was only four or five years old when I remember those days … The kids … and the DEW* line came and then the alcohol came and then they had no time for kids. … [Today] there's so much alcohol and sex mixed that once you get drunk you don't even know if you had sex or not. It's scary right there. (Storyteller, age 53)

*Distant Early Warning (DEW) line refers to the chain of radar stations located across the Arctic coast (42 of which are across the Canadian Arctic), established to protect North America from the Soviet Union during the Cold War (27).

The participating women told stories linking alcohol use to STI and sexual health outcomes and emphasized that programming related to sexual health needed to include discussions around alcohol use and sexual decision-making. This highlights the importance of taking a holistic approach toward programming, in ensuring that the variety of factors that are known to contribute to negative sexual health outcomes within Inuit communities are included in prevention programming. There is a well-established and consistent linkage between alcohol use and STI acquisition (28,29). In addition, within Inuit communities, alcohol both contributes to social and mental health difficulties and is a symptom of existing trauma for individuals and whole communities (30). Alcohol use is a major consideration for future programming to ensure all aspects of health (a holistic view of health) that are included in health promotion programming.

The third major theme, family, was woven through many women's stories. This major theme had three subthemes, one of which was *I tell my children*. Although most of the women stated that they did not learn about sexual health from their parents, they stressed that they are committed to educating their own children. Many women talked about teaching their children, grandchildren and their younger siblings about sexual health. Although it was not necessarily the norm to have open communication about sexual health within their own generation, it is clear that these women recognize the importance of children learning about STI, HIV and sexual health from parents. This comment from a 27-year-old woman illustrates that when she was young she did not receive sexual health information from her parents:

If your parents, like they tell you that you have to go to school in the morning, that's a lesson that you learn from them. Or you've got to go and sweep grandpa's floor, you go and do that. But in our homes, well in my home, using a condom or having safe sex was not something that was said. I never heard my parents talk to me about sex before. (Storyteller, age 27)

This woman acknowledged the value placed on lessons learned from parents and suggested a lesson that was missing in her home was one about safer sex practices. Many participants who were mothers and grandmothers spoke about ensuring that they told their children about sexual health and that they make condoms available for them. This theme emphasizes the family focus of sexual health programming. This sentiment is reflected in the literature, for example, researchers Lys and Reading found that young women want their parents to communicate honestly and frankly about sex with them (31). Youth in the arctic want to learn about sexual health from their parents (31–33), and some studies suggest that parents need support to feel comfortable to do so (9,10,32). Focusing programming on family in order to build the comfort level of discussing topics within the familiy is an important component of future HIV and STI prevention and sexual health promotion programming. Women in this study were committed to sharing what they know with their children, but admittedly they said they need more information and support themselves.

As the storytelling sessions were focused on discussing sexual health, HIV and STIs, the fourth major theme, intimate relationships, was a prominent idea that was discussed. Intimate relationships emerged as a theme throughout women's stories about their observations of other people's relationships, as well as their personal experiences. *Role modelling* was one of the four subthemes (see Table I) that made up the major theme intimate relationships. Here, one woman shares her ideas about being a role model for children and youth with regard to intimate relationships:

So you know, it really comes down to the point that when you want to be a role model to your daughters and granddaughters you have to think twice before you do anything, especially when there's little kids in the house. (Storyteller, age 58)

This theme and subtheme emphasizes the youth-centred family-focused approach for sexual health programming. Literature examining intimate partner violence within Northern communities suggests that when children observe intimate partner violence as a child, they are more susceptible to becoming an offender or victim of intimate partner violence as an adult (34). The idea of positive role models for youth to observe healthy intimate relationships is an important consideration for community sexual health, and it is also inline with Inuit ways of learning through observation.

The fifth major theme, holistic strategies, was made up of nine subthemes, with some subthemes containing nested themes (see Table I). One of these subthemes was *responsibility*. The subtheme *responsibility* emerged from stories discussing where the responsibility of community sexual health promotion and disease prevention education lies. Within the subtheme of responsibility nests several themes regarding who is responsible for sexual health promotion and disease prevention. The participating women told stories of parents, grandparents and other family members, schools, health system and the community all having a hand in community sexual health promotion and disease prevention. *Schools w*ere discussed as a source of sexuality education. Although participating women discussed the current programming that happens within the schools, they felt that more programming was needed:

… going into the schools too with the programming, like there's a program in grade five health, even it started from grade four, about changes in your life for the girls and the boys and talking about how to prevent this type of disease. That helps and I think that needs to be more, also more has to be done at starting into the next school, the high school. (Storyteller, age 61)

In many cases, participating women mentioned several groups who should provide sexual health teaching and support. For example, participating women suggested that the hamlet can host sexual health fairs, parents and Elders can talk to their children and youth, and the health centre can do an STI testing blitz similar to the one the community recently held for diabetes testing. This demonstrates that sexual health is understood to be an issue that should be addressed by a community-wide approach and that there is no one method that will suit everyone in the community. In addition, they noted the need to include those who are sometimes hard to reach, such as youth who do not attend school and men who often do not have community programming and social networks that women have through sewing groups and prenatal groups. The idea that the responsibility for STI and HIV prevention and sexual health promotion should be shared community-wide also speaks to the approach being holistic. Recognizing that the wider community (beyond the health system) has a part to play and indicates a wider definition of sexual health (beyond the absence of disease).

These findings highlight the collective experience of these women's stories. Overall, women who participated emphasized that within their community HIV and STI prevention and sexual health promotion programming needs to take a holistic, community-wide, family-focused and youth-centred approach. Findings indicate that although some programming exists, and individuals are taking it upon themselves to discuss sexual health issues, more support and attention is needed.

CBPR seeks to find a balance between research and action to the benefit of all involved (12). The intent of this project was to initiate action by engaging Inuit women in a dialogue to address the lack of information available to inform programming to improve the sexual health of Inuit women, their families and their communities. This study contributes knowledge on community HIV and STI prevention and sexual health promotion within Inuit communities; however, it is both the process and results that are helpful in guiding future research and programming. The incorporation of IQ into the research framework has shown to be a promising approach for Inuit CBPR. The importance of taking time to meet and spend time with participants and having a relationship with the community cannot be emphasized enough. The pre-existing relationship between this community and the primary researcher meant that the trust needed to undertake CBPR was already established. Because there has been very little HIV prevention research conducted in Inuit communities, this project has gleaned important information to add to a small body of existing literature.

## Conclusion

This study was grounded in IQ and the strengths-based understanding that Inuit women are experts in their own lives. It is their ideas and insights that need to be sought as the basis for STI and HIV prevention and sexual health promotion programming for themselves and their families in their own communities. The findings from this study are derived directly from women who live in the region with some of the highest STI rates in Canada (4). This study contributes to the growing body of community-based literature that considers the perspectives of the people living in these communities (1,2,11,20,21). The narratives emerging from this study create linkages between what is happening in the daily lives of women and families and the overwhelming epidemiological statistics. These women's stories illustrate what is needed to address sexual health promotion and disease prevention on the ground in their community and to reveal that this programming needs to be holistic, family-focused, youth-centered and take a community-wide approach.

Direction for future research and health programming can also be found in other Inuit and northern sexual health research taking place in Canada and circumpolar regions (1,3,9,10,33,35–37). This study not only adds to the growing body of literature regarding Inuit sexual health, but also provides an example of Inuit research methodologies. With the continuous development and ever-growing research interest in the Canadian North, across disciplines, studies such as this one, as well as the work done across the North (38–43) provide examples and tools that continue to enable Inuit communities to determine and drive their own research and build community capacity to do so.

## Similar publications

This article is based on research completed for the primary researcher's Master's thesis.
